# Better long-term speech outcomes in stroke survivors who received early clinical speech and language therapy: What’s driving recovery?

**DOI:** 10.1080/09602011.2021.1944883

**Published:** 2021-07-02

**Authors:** Sophie Roberts, Rachel M. Bruce, Louise Lim, Hayley Woodgate, Kate Ledingham, Storm Anderson, Diego L. Lorca-Puls, Andrea Gajardo-Vidal, Alexander P. Leff, Thomas M. H. Hope, David W. Green, Jennifer T. Crinion, Cathy J. Price

**Affiliations:** a Wellcome Centre for Human Neuroimaging, UCL Queen Square Institute of Neurology, London, UK; b Faculty of Health Sciences, Universidad del Desarrollo, Concepcion, Chile; c Institute of Cognitive Neuroscience, University College London, London, UK; d Department of Brain Repair and Rehabilitation, UCL Queen Square Institute of Neurology, London, UK; e Department of Experimental Psychology, University College London, London, UK

**Keywords:** Aphasia, speech and language therapy, lesion site, patient-reported outcome measures, observational study

## Abstract

Establishing whether speech and language therapy after stroke has beneficial effects on speaking ability is challenging because of the need to control for multiple non-therapy factors known to influence recovery. We investigated how speaking ability at three time points post-stroke differed in patients who received varying amounts of clinical therapy in the first month post-stroke. In contrast to prior studies, we factored out variance from: initial severity of speaking impairment, amount of later therapy, and left and right hemisphere lesion size and site. We found that speaking ability at one month post-stroke was significantly better in patients who received early therapy (*n* = 79), versus those who did not (*n* = 64), and the number of hours of early therapy was positively related to recovery at one year post-stroke. We offer two non-mutually exclusive interpretations of these data: (1) patients may benefit from the early provision of self-management strategies; (2) therapy is more likely to be provided to patients who have a better chance of recovery (e.g., poor physical and/or mental health may impact suitability for therapy and chance of recovery). Both interpretations have implications for future studies aiming to predict individual patients’ speech outcomes after stroke, and their response to therapy.

## Introduction

The goal of this study was to investigate whether recovery of speaking ability was related to the provision, and/or amount, of clinical speech and language therapy received in the first month after stroke, after controlling for initial severity, amount of later therapy, handedness, comprehension ability, time post-stroke and lesion size and site. Prior studies have yielded inconsistent conclusions about the benefit of early speech and language therapy for post-stroke aphasia (see Supplemental Material Section 1 for a full review). Even when positive effects of therapy are reported, there are concerns that these effects are weak, not clinically meaningful, and without generalization or maintenance (Brady et al., 2016). For example, in several pilot studies into early therapy, Godecke et al. (2012, 2014) reported a significant benefit of higher intensity therapy in the first month after stroke that was lost at 6-month follow up. More recently, the same authors found no significant benefit of higher-intensity therapy in a randomized controlled trial (Godecke et al., 2020). This may be because establishing true therapy effects requires strictly controlled trials where patients who did and did not receive therapy are matched for other known predictors of outcome such as initial symptom severity, lesion site and size (Watila & Balarabe, 2015). The latter was not possible in prior studies because neuroimaging data were not available.

The current study constituted a retrospective analysis of how speaking outcomes in stroke survivors with aphasia differed in those who did and did not receive non-experimental, clinical speech and language therapy, administered by health services, in the first month after stroke; and whether any benefit of therapy was enhanced by the number of hours received. In contrast to previous studies, we investigated (1) clinical therapy rather than experimental therapy and (2) long-term maintenance of the therapy gains. We controlled for (i) initial severity in the first week after stroke; (ii) intervening therapy between one month and one year and (iii) left and right hemisphere lesion size and site, and were still able to keep participant numbers high (total *n* = 143) because stroke survivors were selected from a much larger cohort of patients who have participated in the Predicting Language Outcome and Recovery After Stroke (PLORAS) study (Seghier et al., 2016). Critically, the selected patients had reported, via questionnaires, how many hours of therapy they had received and when this therapy took place.

**Figure 1.**
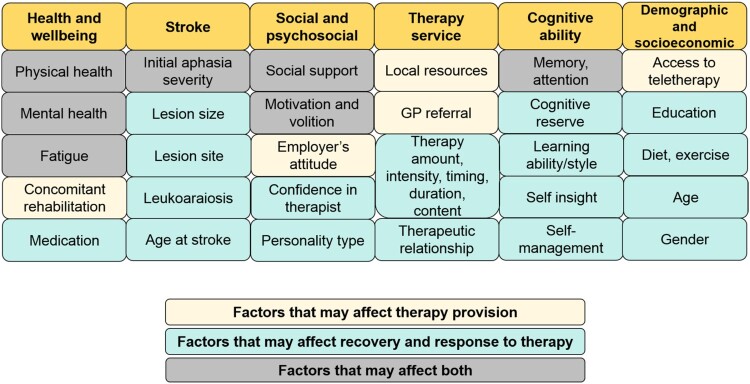
Factors that may affect the provision of speech therapy, and recovery and response to therapy.

By controlling for multiple variables that are known to affect outcome and recovery of speech after stroke, we aimed to sensitize our study to the effects of early clinical therapy. If recovery is better in patients who (A) did, versus did not, receive early therapy and (B) had greater versus fewer hours of early therapy, we could infer that early therapeutic activities had a beneficial effect. If only (B) is true, we could infer that early therapy had a beneficial effect when the hours of therapy were sufficiently high. If only (A) is true, we could infer that either the patients benefitted from the early provision of self-managed relearning strategies, or that therapy was only available or suitable for patients who had the most chance of recovery (e.g., those with better physical and/or mental health); see Figure 1. Both interpretations have important implications for understanding how speech and language recovery, and the effect of therapy, can be predicted in the future.

We assessed the effect of early clinical therapy on speaking ability using scores from both patient-reported outcome questionnaires and objective behavioural assessments conducted by speech and language therapists. The two major advantages of using patient-reported outcome measures (PROMS) are: (i) the impact of therapy is assessed from the patient’s perspective; and (ii) the time points at which the effect of therapy was assessed (i.e., one month and one year post-stroke) were controlled across patients. The disadvantage of PROMS is that they are acquired retrospectively and therefore depend on the patients’ memories of, and insight into their ability to communicate at different times post-stroke. Although we excluded patients whose memory of therapy time was vague or inconsistent, a false negative result (i.e., the absence of a therapy effect) could occur if inter-patient variability in memory accuracy was greater than a true therapy effect. In contrast, false positives could result if patients were more likely to perceive an improvement over time when they received more, rather than less, therapy. To avoid such bias, we also measured speaking abilities with objective, behavioural tests (Naming, Repetition, and Spoken Picture Description) at the time that patients entered the PLORAS study. The disadvantages of our objective scores are: (i) they were obtained at different time points post-stroke (months to years), depending on when the patient entered the study and, (ii) performance on any one task depends on many interrelated perceptual, cognitive and motor functions (the task impurity problem), therefore isolating speaking outcomes requires us to dissociate variance that is related or unrelated to speech production: e.g., auditory word repetition from auditory word comprehension and object naming from object recognition; dissociations we were able to address in our investigation (for details, see section on Comprehensive Aphasia Test in Methods).

A robust outcome would be one where an effect of early therapy on the ability to speak was observed in both PROMS and objective assessments after controlling for time post-stroke at test, and other cognitive abilities. In addition, unlike prior studies, we used left and right hemisphere lesion size as an estimate of the brain’s capacity to recover (Benghanem et al., 2019; Meier et al., 2019; Thye & Mirman, 2018). If patients who did not receive early speech and language therapy had larger lesions, worse speaking outcomes may have been the consequence of less capacity for recovery (because of the larger lesions) rather than the absence of therapy. In addition, patients with larger lesions may have had less time and cognitive resources for speech and language therapy because of the presence of concomitant impairments that needed a variety of interventions, such as physiotherapy and occupational therapy.

## Materials and methods

The study was approved by the London Queen Square Research Ethics Committee, and all participants (or consultees) gave written informed consent to participate.

### Patient selection

Our participants were 143 stroke survivors, with no other neurological or psychiatric conditions that might influence their speech and language abilities. All were native English speakers who (i) had a left hemisphere lesion that was larger than 1cm^3^, (ii) had fully completed our in-house Aphasia Recovery and Therapy Questionnaire, (iii) were severely or moderately aphasic one week after their stroke (defined as unable to produce speech, or only able to produce single words), and (iv) were assessed in full with the Comprehensive Aphasia Test (CAT). Details of these 143 participants can be found in Table 1.

**Table 1.** Patient characteristics.

		Severe		Moderate	
		No Therapy	Therapy^▴^	No Therapy	Therapy^▴^
Group size		49	59	15	20
Early therapy amount (hours)	Mean (SD)	0	8 (6)	0	9 (8)
	Range	-	1–20	-	2–30
Later therapy amount (hours)	Mean (SD)	33 (29)	37 (35)	10.9 (12)	38 (41)
	Range	0–100	0–220	0–32	0–191
Sex	Male (%)	36 (73)	43 (73)	13 (87)	15 (75)
	Female (%)	13 (27)	16 (27)	2 (13)	5 (25)
Pre-stroke handedness	Right (%)	47 (96)	49 (83)	15 (100)	14 (70)
	Left (%)	2 (4)	6 (10)	0 (0)	2 (10)
	Ambidextrous (%)	0 (0)	4 (7)	0 (0)	4 (20)
Age at stroke (years)	Mean (SD)	56 (14)	57 (13)	61 (9)	57 (11)
	Range	33–85	30–82	45–73	37–70
Years post-stroke of CAT	Mean (SD)	4 (4)	4 (4)	4 (3)	4 (4)
	Range	0–23	1–17	1–9	1–17
Years post-stroke of PROM*	Mean (SD)	5 (4)	5.5 (5)	4 (3)	5 (5)
	Range^	1-16.5	0.9-24	1–9	1-16.5
Lesioned hemisphere	Left (%)	42 (86)	50 (85)	10 (66)	18 (90)
	Both (%)	7 (14)	9 (15)	5 (33)	2 (10)
Left hemisphere lesion size (cm^3^)	Mean	70 (70)	73 (59)	32 (37)	43 (55)
	Range	3–355	1–235	2–119	1–194
One week understanding◊	None (%)	14 (29)	19 (32)	1 (7)	0 (0)
	Words (%)	14 (29)	10 (17)	4 (27)	8 (40)
	Sentences (%)	5 (10)	13 (22)	7 (47)	7 (35)
	Normal (%)	15 (31)	16 (27)	3 (20)	5 (25)

^▴^ Early therapy data are self-reported retrospectively when patients enter the PLORAS study; see Table 2 for questions asked.

*Time post-stroke of PROM is missing for: 8 in the No Therapy-Severe group, 8 in the Therapy-Severe group, 6 in the No Therapy-Moderate group, and 4 in the Therapy-Moderate group.

^8 patients had not reached one-year post-stroke when the one-week and one-month data were collected. The time post-stroke data reflects the time the one-year data were collected.

◊One-week understanding scores are missing for: 1 in the No Therapy-Severe group and 1 in the Therapy-Severe group.

Our sample did not include patients who were medically unwell (e.g., in a coma, or mechanically ventilated) in the first week after their stroke because the inability to produce speech in these patients was not necessarily related to the presence of aphasia and these patients would not have been well enough to engage in, or benefit from, therapy. This is crucially different from the patients in the Severe category, who were conscious, and physically capable of attempting to speak – but could not produce any words, due to aphasia (and/or dysarthria/apraxia). We also excluded patients with Mild initial difficulties (defined as being able to generate short sentences in the first week after stroke) because, of the 63 patients we identified with Mild initial severity: (i) only 3 (5%) received more than 4 h of early therapy (within the first month) and (ii) only 12 (19%) of those who did not receive any early therapy failed to recover to normal within a year post-stroke. Therefore, we did not have sufficient data to assess the effect of therapy in these patients.

Patients were selected from the PLORAS database; see Seghier et al. (2016) for details. The PLORAS study recruits from a broad variety of settings, including approximately 70 English hospitals via the National Institute for Health Research (NIHR) Clinical Research Network, stroke clubs, and speech and language therapy clinics, as well as self-referrals and word-of-mouth recommendations. As such, each patient’s experience of rehabilitation differs due to regional variation in service provision. The benefit of this is that the data accurately reflects typical clinical speech and language therapy provision around England (see below for more detail). The disadvantage is that we were unable to control for therapy type and approach.

### Speech and language therapy

Using an in-house questionnaire administered when patients enter the PLORAS study, we asked each patient: (i) how many hours of speech and language therapy they had received, (ii) when speech and language therapy was received (start and end points and frequency) and (iii) therapy activities (see Table 2). Patients are supported in providing this information by a speech and language therapist, who facilitates both the patients’ understanding of the questions, and their responses, and helps them to differentiate direct language therapy from other typical therapist input delivered during the acute stage post-stroke; e.g., assessment, dysphagia management, or information and monitoring.

**Table 2.** Questions asked to participants about their speech and language therapy.

Have you received any speech and language therapy since your stroke? **Yes/No If yes:** When did therapy start When did therapy end? How often did you do therapy? (e.g., one hour every week) What sort of activities did you do in therapy?

The speech and language therapy received by our patients can be described as “clinical” (i.e., part of routine care) rather than “experimental” (i.e., delivered as part of a research project). Speech and language therapy provision in the UK is guided by NICE (National Institute for Health and Care Excellence) clinical guidelines, which recommend screening for communication impairments within 72 h of stroke (NICE, 2013). If speech therapy is required, it should be provided in 45 min-long sessions (or less, for those who cannot tolerate this amount), for a minimum of 5 days per week. Further rehabilitation can then be offered for those who can participate and continue to make functional gains. There are no additional specific recommendations for the timing, amount, intensity and duration of therapy. If communication difficulties persist at 6-month or annual review, patients can be re-referred to speech therapy services, and offered further treatment if they have the potential for functional improvement. Clinical aphasia therapy typically comprises two broad approaches: (1) functional, and (2) impairment-based, targeting expressive and/or receptive language as required. All therapy aims to reduce impairment severity, increase communicative ability, and/or implement alternative methods of communicating, as well as to provide information and support to both the patient and the carer or communication partner. Impairment-based therapy may target speech sounds, single words, or sentences, and therapists provide support and guidance through strategies such as modelling and cues.

As our patients were recruited from a broad range of geographical locations and clinical settings across England, we were not able to control for therapy content or approach in our sample, and we recognize that there are many ways a speech therapist could have contact with a patient which do not include language therapy; e.g., assessment time or dysphagia management. Nonetheless, because all our patients had difficulties producing speech in the first week after their stroke, we were able to assume that the clinical therapy they received included some therapy or strategies aimed to improve their speaking difficulties. In summary, it is highly likely that therapy included activities to improve speaking ability, although the actual amount of therapeutic input may be over-estimated.

**Figure 2.**
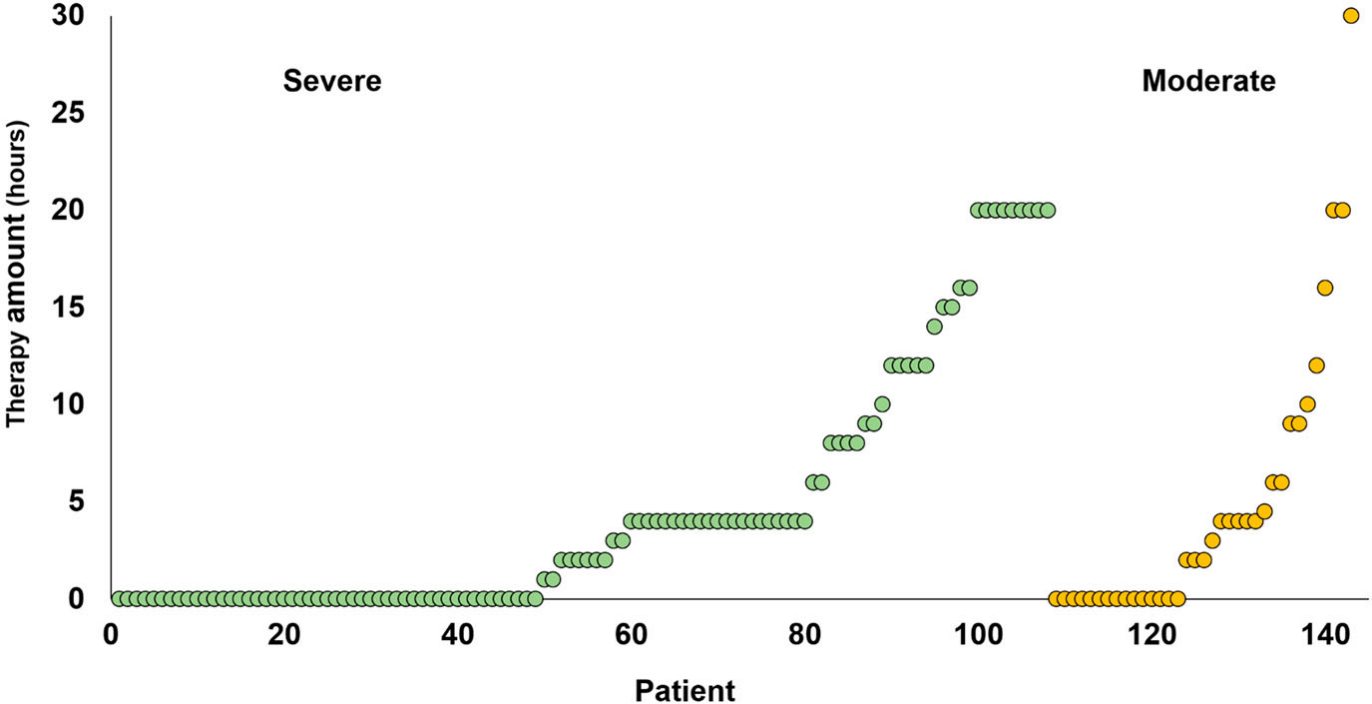
Amount of early therapy (hours) reported by each patient, according to whether the initial severity of symptoms was Severe or Moderate.

The amount of therapy received by each of our patients can be seen in Figure 2, and we observe that the majority of our patients received less than currently recommended (3-4 h per week) (NICE, 2013). Nevertheless, it might be close to the actual amount of therapy typically received, given that recent national audit data shows that the target amount of therapy is frequently unmet due to time spent on administration (therapy planning and documenting), and patient factors (medical instability and fatigue) (Clarke et al., 2018). There may also be a regional disparity in the amount of therapy provided by the National Health Service in the UK due to differences in service structure (SSNAP, 2017). Our study capitalized on this regional variability to reveal the influence of therapy.

An advantage of clinical therapy, over experimental therapy, is that clinical therapy is tailored around the patient’s personal needs, goals and learning style. A natural disadvantage follows, namely that therapy differs for everybody, even if the overall goal of improving speaking remains the same. Another disadvantage is that the amount of clinical therapy received is typically lower than that reported in experimental trials. Although fewer hours of therapy could have desensitized our study and resulted in false negative results, (i) this was not the case and (ii) by analysing effects of what is typically received, rather than ideally received, we reduce the gap between what is often researched, and what is actually being delivered. Focusing retrospectively on clinical therapy also allows us to assess how well patients recovered after receiving no speech and language therapy, without facing ethical issues related to prospectively withholding therapy.

### Patient-reported outcome measures of speaking ability

Participants retrospectively and categorically rated their communicative ability at three fixed time points after stroke onset: one week, one month and one year. For each time point, they rated their ability to speak. We categorized these ratings as: Severe when patients were unable to produce any voluntary speech due to aphasia, dysarthria and/or apraxia, as opposed to a lack of consciousness; Moderate when patients were able to produce words but not sentences; Mild when they could produce lexically meaningful short sentences; or Normal when they did not report an impairment. Speech therapists support and encourage patients to provide more details where necessary, to ensure a correct understanding of their difficulties (for example, differentiating fluent aphasia from non-fluent aphasia). Specific types of speech production impairments, e.g., word-finding difficulty versus articulatory difficulty were not differentiated in the patient-reported outcome measure but were examined in our objective assessments (see below). Carers were encouraged to provide their own report of patients’ abilities and therapy, either to supplement the patient’s report, or to substitute it if the patient had memory or other cognitive impairments that prevent them from providing sufficient details. Our sample of 143 patients did not include any patients where the carer and patient gave inconsistent reports (15 had already been excluded for this reason).

‘Improvement’ from one time point to another was denoted by any change in category (Severe → Moderate → Mild → Normal), as this represents a clinically meaningful improvement, being both functionally important, and indicating a reduction in impairment. The degree of improvement was calculated by assigning each patient a score of 0 (no change in category), 1 (from Severe to Moderate, or from Moderate to Mild), 2 (from Severe to Mild, or from Moderate to Normal), or 3 (from Severe to Normal).

### Comprehensive Aphasia Test

All patients were assessed with an objective language and cognitive assessment, the Comprehensive Aphasia Test (CAT) (Swinburn et al., 2004). The CAT is a fully standardized test battery, which consists of a total of 27 different tasks. The current study selected 3 speaking measures from the CAT to evaluate therapy effects: (1) repetition, a composite measure of a patient’s ability to repeat heard words (e.g., *plant*), non-words (e.g., *trimpy*), complex words (e.g., *president*), sentences and digit strings; (2) spoken naming, a composite measure of object naming, action naming and verbal fluency, and (3) spoken picture description, which measures connected speech, including appropriateness of information-carrying words, grammatical accuracy, syntactic variability and speed of production. Additionally, we controlled for the impact of non-speech (perceptual and semantic) impairments on the ability to perform our speaking tasks by factoring out performance on two other CAT tasks: (4) auditory word comprehension, which measures the patient’s ability to match pictures to a heard word, in the presence of phonological and semantic distractors. This was included because poor auditory word comprehension will affect the ability to repeat words; and (5) semantic memory, which measures the ability to perceive pictures and identify semantic links (e.g., monkey and banana). This was included because poor performance on this task will affect the ability to name objects and describe pictures.

To compare performance on different tasks, raw scores are converted (through a non-linear transformation, see the CAT manual for more details) into T-scores, which represent how well the patient performed relative to an independent sample of patients with aphasia. A T-score of 50 indicates the mean of the patient sample used to standardize the CAT, whereas a T-score of 60 represents one standard deviation above the mean. Lower scores indicate poorer performance. T-scores can be compared using parametric statistics because they are normally distributed.

### Statistical analysis of therapy effects on behaviour

All statistical analyses of recovery related to therapy were performed in IBM SPSS Statistics (version 25.0), using 2-tailed *p* values. Two different types of regression analysis were used for the two different outcome measures. Binary logistic regression was used to assess the effect of therapy (i.e., a binary outcome of improvement or no improvement) on patient-reported outcome measures between one week and one month, and between one month and one year. Multiple linear regression was used to assess the effect of therapy on the linear CAT Naming, Repetition, and Spoken Picture Description scores. Each of these 5 analyses were performed twice (10 analyses in total) with therapy either treated as a binary variable (presence versus absence of therapy in the first month after stroke) or a continuous variable (number of therapy hours in the first month after stroke).

All 10 analyses factored out variance of no interest by including the following covariates: initial severity (Severe or Moderate), age at stroke, handedness, left hemisphere lesion size and right hemisphere lesion size. In addition, amount of later therapy (between one month and one year) was factored out of all analyses except improvement between one week and one month. For the analyses using CAT scores, we also factored out time post-stroke that the CAT was administered, semantic matching scores from the CAT (to control for object recognition and semantic memory) and auditory word comprehension scores from the CAT (to control for auditory perception and comprehension).

We are confident that there was minimal collinearity in the data because none of the analyses yielded Tolerance values of less than 0.1, nor Variance Inflation Factor (VIF) values that were greater than 10. Moreover, none of our independent variables have correlation values greater than 0.7 (see Supplemental Figure 1).

### Lesion analyses

High-resolution (1 mm x 1 mm x 1 mm), whole brain T1-weighted structural brain images were acquired for all patients on research-dedicated scanners at the Wellcome Centre for Human Neuroimaging and the Birkbeck-UCL Centre for Neuroimaging. The MRI scanners used were all from Siemens Healthcare (Erlangen, Germany): 40 patients were imaged on a 3 T Trio scanner, 42 on a 3 T Prisma scanner, 23 on a 1.5 T Sonata scanner, 37 on a 1.5 T Avanto scanner, and 1 on a 3 T Allegra scanner.

Using standard procedures within SPM software (Wellcome Centre for Human Neuroimaging, London, UK; https://www.fil.ion.ucl.ac.uk/spm/), running in MATLAB environment (2018a Mathworks, Sherbon, MA, USA), each T1-weighted image was spatially normalized (to the MNI template) and converted into a quantitative assessment of structural abnormality that is independent of the scanner used (Seghier et al., 2008). At each voxel, this “fuzzy lesion image” encodes the degree of abnormality on a continuous scale from 0 (completely normal) to 1 (completely abnormal) relative to normative data from a sample of 64 neurologically-intact controls. To delineate the lesions, estimate lesion volume, and generate lesion overlap maps, each fuzzy lesion image was thresholded into a “binary lesion image” (i.e., lesion/no lesion). The abnormality threshold used was 0.3 (U value, on the 0–1 scale described above), as recommended in Seghier et al. (2008), after optimization from data collected on our scanners.

To investigate whether lesion site differed in patients who did and did not receive early clinical therapy, we entered the whole brain fuzzy images into a voxel-based morphometry (VBM; (Ashburner & Friston, 2000)) analysis performed in SPM12, using the general linear model. Like other voxel-based lesion symptom mapping (VLSM) methods, VBM searches the whole brain for voxels where local brain structure varies with a symptom or other variable of interest (here the presence or absence of therapy or the amount of therapy). Many previous studies have demonstrated the sensitivity of VBM/VSLM but there are also limitations to the approach (Gajardo-Vidal et al., 2018; Ivanova et al., 2021; Zhang et al., 2014). For example, the effect of damage to one area may depend on the presence or absence of damage to another area. Understanding such combinatorics requires multivariate lesion analyses on large samples of patients but as these methods are still in their infancy and have numerous interpretation problems (Ivanova et al., 2021) they were not used in the current study.

The fuzzy images used in our VBM analysis index the degree to which each voxel differs from the normal range and therefore avoids reliance on binary cut-offs (Gajardo-Vidal et al., 2018). The statistical model for our VBM was an ANOVA with four different independent groups of fuzzy images, with unequal variance: (1) Severe initial severity and early clinical therapy (*n* = 59); (2) Severe initial severity and no early therapy (*n* = 49); (3) Moderate initial severity and early clinical therapy (*n* = 20); and (4) Moderate initial severity and no early therapy (*n* = 15). The statistical contrasts computed the main effects of Therapy versus No Therapy (across initial severity), Severe versus Moderate initial severity (across therapy groups), and the interaction between therapy and initial severity.

To investigate whether lesion site differed in patients who received more versus less therapy, we repeated the four-group analysis again, this time including two covariates (i.e., ANCOVA): (1) number of hours of early therapy; and (2) number of hours of later therapy. The results from both analyses (ANOVA and ANCOVA) are reported at a significance level of *p* < 0.05 corrected for multiple comparisons in extent or in height within our left hemisphere search volume, defined as all the voxels (36,037 =  288.3 cm^3^) that were damaged in at least 10 of the 143 patients in our sample.

## Results

When early clinical therapy was treated as a binary measure, we identified two significant effects (Table 3): compared to no therapy, patients who received early clinical therapy had better (i) patient-reported improvement at one month and (ii) Naming scores when objectively assessed with the Comprehensive Aphasia Test (CAT). A significant effect of hours of later therapy was also seen on Naming (*p* = 0.036) at the time of the CAT assessment.

**Table 3.** Outcomes in 143 patients who reported (i) whether they did versus did not receive early therapy and (ii) varying amounts of early therapy.

	Outcome measure	R^2^	Model significance	Standardized Beta	*P* (2 tailed)
*Binary analyses: Early therapy or none*					
1	Patient-reported improvement at one month	.203	.002	2.256	.045
2	Patient-reported improvement at one year	.186	.020	1.779	.213
3	Naming	.557	.000	.158	.013
4	Repetition	.462	.000	.109	.112
5	Spoken Picture Description	.499	.000	.043	.517
*Continuous analyses: hours of early therapy*					
6	Patient-reported improvement at one month	.172	.008	1.018	.551
7	Patient-reported improvement at one year	.222	.005	1.117	.045
8	Naming	.540	.000	.074	.245
9	Repetition	.460	.000	.099	.147
10	Spoken Picture Description	.499	.000	.044	.500

Covariates of no interest: Initial severity (Severe or Moderate), handedness, age at stroke, left hemisphere lesion size, right hemisphere lesion size, (all models), amount of later therapy (all models except one month), semantic memory CAT scores, auditory word comprehension CAT scores, and time post-stroke of CAT (for Naming, Repetition and Spoken Picture Description).

When early clinical therapy was treated as a continuous measure, we also found that patient-reported improvement at one year was positively related to the number of hours of early therapy. This was observed across the whole sample (*n* = 143, see Table 3). These results remained significant when the one patient who received an exceptional amount of early therapy (30 h, see Figure 2 in the Moderate initial severity group) was removed from the analyses. Furthermore, the results did not change when patients with outlying scores on Naming (*n* = 6) and Repetition (*n* = 4) were also removed from the relevant analyses (see Table 4). There were no significant improvements related to later therapy in the analyses using continuous measures.

**Table 4.** Outcomes in patients who reported (i) whether they did versus did not receive early therapy and (ii) varying amounts of early therapy, with outliers removed from analyses.

	Outcome measure	*R*^2^	Model significance	Standardized Beta	*P* (2 tailed)
*Binary analyses: Early therapy or none*					
1	Patient-reported improvement at one month	.216	.001	2.455	.030
2	Patient-reported improvement at one year	.183	.023	1.765	.220
3	Naming	.446	.000	.189	.009
4	Repetition	.414	.000	.120	.101
5	Spoken Picture Description	.502	.000	.048	.467
*Continuous analyses: hours of early therapy*					
6	Patient-reported improvement at one month	.187	.004	1.039	.236
7	Patient-reported improvement at one year	.218	.006	1.117	.046
8	Naming	.419	.000	.074	.312
9	Repetition	.415	.000	.125	.087
10	Spoken Picture Description	.504	.000	.068	.303

Covariates of no interest: same as above.

Outliers: 1 outlier with 30 h of therapy removed from all models. 6 patients with outlying Naming scores removed from Naming analyses. 4 patients with outlying Repetition scores removed from Repetition analyses.

**Figure 3.**
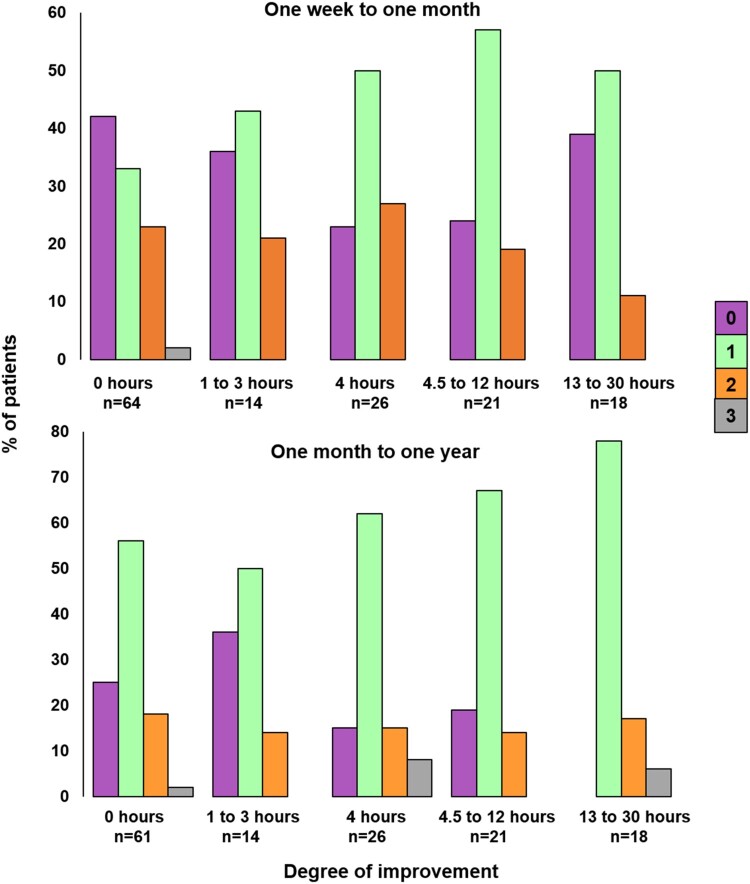
Degree of improvement from one week to one month, and one month to one year, according to five different patient-reported therapy amounts. The colour of the bar indicates the degree of improvement: Purple =  no change in score, green =  a change of one (e.g., Severe to Moderate, Moderate to Mild), orange =  a change of two (Severe to Mild, Moderate to Normal) and grey =  a change of three (Severe to Normal).

The differing one-month and one-year outcomes after varying therapy amounts are illustrated in Figure 3. At one month (upper part of Figure 3), the number of patients with no improvement is lower for those who reported receiving more than 4 h of therapy, but it does not continue to decrease as the number of hours of therapy increases. In contrast, at one year (lower part of Figure 3), the number of patients who improved is higher after 13–30 h of early therapy compared to 4–12 h of early therapy.

**Figure 4.**
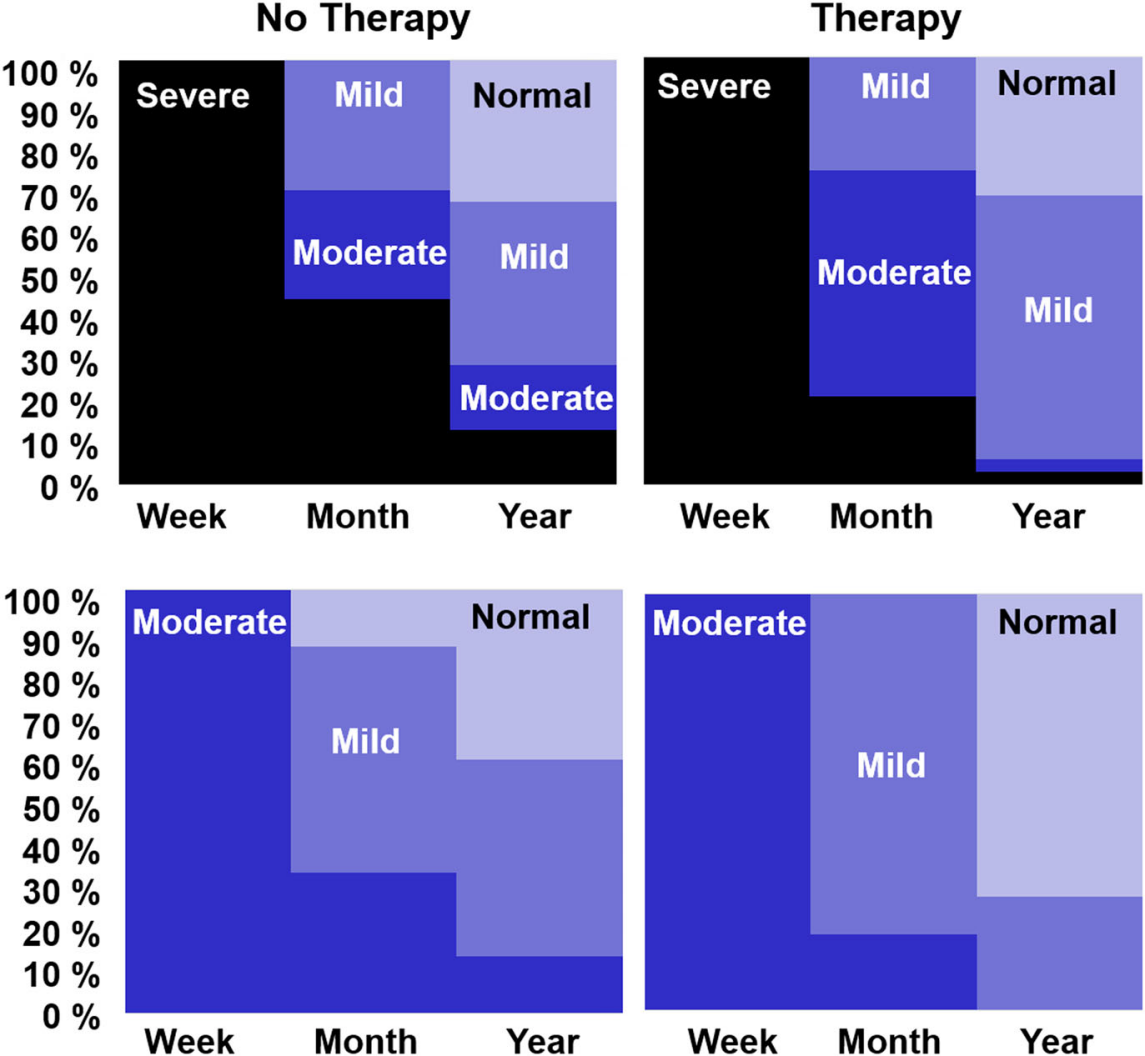
Degree of improvement for four patient groups with either Severe or Moderate initial severity, and who reported either no early therapy or more than 4 h of early Therapy.

Figure 4 illustrates patient outcomes from one week through to one year in four different groups of patients who either had: (1) Severe initial severity and early clinical therapy (*n* = 39), (2) Severe initial severity and no early clinical therapy (*n* = 34), (3) Moderate initial severity and early clinical therapy (*n* = 15), or (4) Moderate initial severity and no early clinical therapy (n = 11). To maximize between-group differences in the amount of early therapy, we excluded 14 patients who received 1–3 h of early therapy (in first month). To focus on the influence of early therapy, we also excluded 30 patients who received 50 h or more of later therapy (between one month and one year). After these exclusions, the average number of hours of early therapy was: 9 (Severe group, with therapy) and 7 (Moderate group with therapy), compared to zero in both the groups with No Therapy. The average amount of later therapy was 21.7 (Severe group with early therapy), 20.5 (Severe group with no early therapy), 22.5 (Moderate group with early therapy) and 10.9 h (Moderate group with no early therapy). It was not possible to match later therapy in the Moderate groups more closely because of the smaller group size.

**Figure 5.**
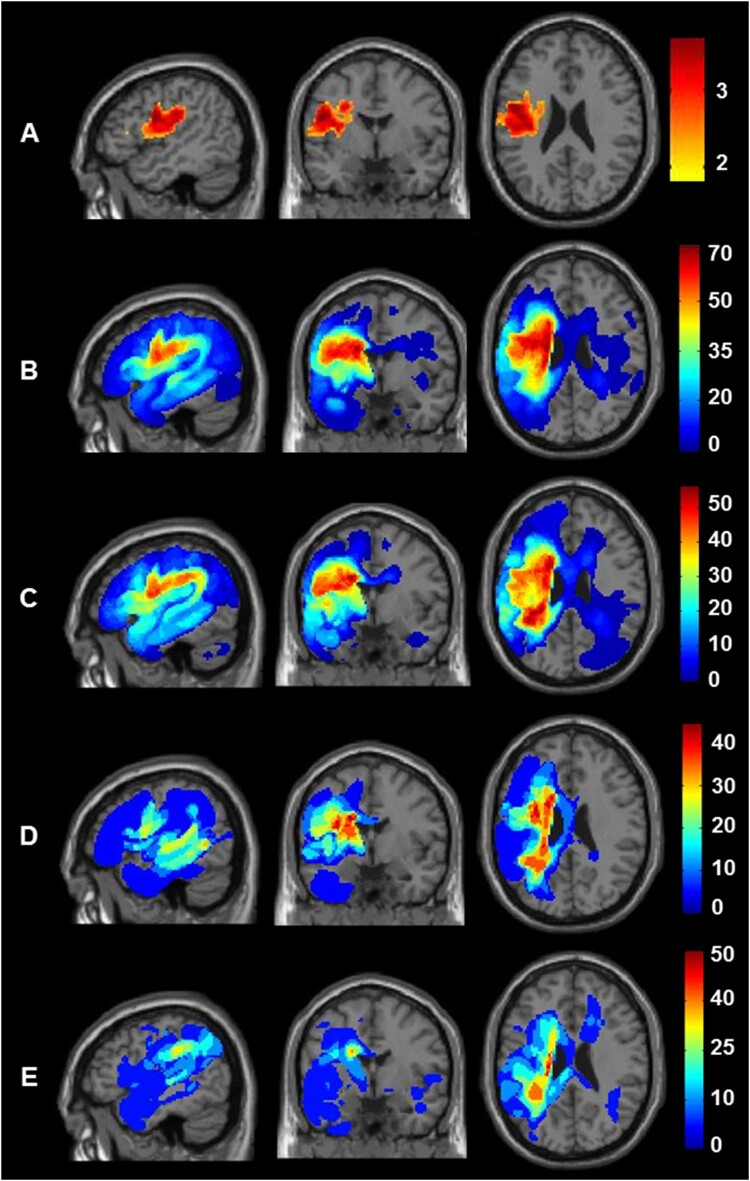
Comparison of lesion sites across the four different groups. Lesion site results after assigning the 143 patients to four groups according to initial severity (Severe versus Moderate) and the provision of therapy (Yes versus No). Top row (A) shows the brain regions where damage was significantly greater in those with Severe versus Moderate initial severity. The sagittal, coronal and axial slices are at the co-ordinates of the most significant group difference (−48, −2, +24). Rows B to E show the lesion overlap maps (showing the frequency of damage) for the four groups at the same co-ordinates. From top to bottom, B) Patients with Severe initial severity who received Therapy (*n* = 59); C) Patients with Severe initial severity who did not receive therapy (*n* = 49); D) Patients with Moderate initial severity who received Therapy (*n* = 20); E) Patients with Moderate initial severity who did not receive therapy (*n* = 15). The colour scale indicates percentage of patients for each group.

Finally, the lesion analyses show that poorer outcomes in patients who did not receive early therapy could not be explained by (A) larger lesion sites or (B) more damage to regions required for recovery because global and local brain structure did not vary between therapy groups; nor did it depend on the number of hours of early therapy received. The same analyses were, nevertheless, highly sensitive to two other effects. First, in both analyses (with and without covariates), patients who had Severe initial severity had more extensive damage to the left premotor cortex and underlying white matter (see row A of Figure 5). This was significant, after correction for multiple comparisons within the left hemisphere search volume in both height and extent (peak Z score =  4.5 at MNI co-ordinates [−48, −2, +24], with 3679 voxels at *p* < 0.001 uncorrected). *Second*, in the analysis with early and later therapy added as covariates, there was a significant effect of later therapy: Patients who received more hours of later therapy had more extensive lesions in the white matter beneath the left superior temporal gyrus. This was significant, after correction for multiple comparisons within the left hemisphere search volume in both height and extent (peak Z score =  4.34 at MNI co-ordinates [−42, −40, +8], with 384 voxels at *p* < 0.001 uncorrected). The latter result suggests that patients with damage involving this part of the left temporal lobe required, or were offered, more therapy.

## Discussion

In a review of the literature comparing the effect of speech and language therapy on speaking outcomes, we found no evidence that therapy in the first month after stroke resulted in long-term behavioural gains (see Supplemental Material, Section 1). The majority of these studies investigated experimental therapy, in varying amounts – but even the study by Bowen et al. (2012), which closely resembled our study by investigating a relatively little amount of early clinical therapy, did not find a significant benefit of therapy. We hypothesized that these studies might not have been able to detect the beneficial effect of therapy because of their small sample sizes, or because they did not control for the multiple non-therapy factors that are known to influence recovery, including initial severity, left and right hemisphere lesion size and site, age at stroke, therapy received between the intervention period and the follow-up time point, and other neuropsychological impairments. When we controlled for all these factors, we found evidence for both short- and long-term speaking improvements in those who received early therapy.

In the Introduction, we distinguished between three different scenarios. The first is when better recovery is observed in patients who (A) did versus did not receive early clinical speech and language therapy (i.e., binary therapy analyses), and (B) had greater versus fewer hours of early therapy (i.e., continuous therapy analyses). We did not find an effect of both (A) and (B) on the same outcome measure, therefore this scenario is not relevant here.

The second scenario is when only (B) is true (better recovery in patients who had greater versus fewer hours of early therapy). The positive relationship we found between the number of hours of early therapy and patient-reported outcome at one year post-stroke may be explained by therapy being more beneficial when the amount delivered is sufficiently high. At one month post-stroke, proportionally more patients recovered if they received 4–12 h of therapy compared to 0–3 h of therapy (see Figure 3) but this did not reach significance.

The third scenario is when only (A) is true (i.e., better recovery in patients who did, versus did not, receive early clinical speech and language therapy). This was the case for one month outcomes, and Naming ability at the time of the CAT.

Below, we discuss alternative interpretations of our findings in relation to these two scenarios, along with the limitations of our study, and new directions for future studies.

### Interpreting the long-term benefits in those who received early therapy

We offer two different speculative explanations that might individually, or in combination with one another, explain our findings. First, a few hours of early therapy in the first month after stroke may be sufficient to (i) impart learning strategies that can be used by the patient with their families to facilitate recovery, (ii) motivate patients to self-manage extensive practice and training of functional skills, and/or (iii) encourage and facilitate patients to join communication support groups that positively influence their recovery. Such activities could explain why we found a significantly greater improvement for patient-rated outcomes from one month to one year (i.e., after the early therapy period) when patients received more early therapy. In other words, we are suggesting that the patients who received therapy may have continued to practise and benefit from the self-management strategies that they were taught early post-stroke, compared to those who did not receive therapy and who had little/no guidance for recovery. Examples of self-management strategies that our patients described include (i) reading aloud from newspapers or magazine articles, (ii) identifying personally-relevant key words around the home, and increasing their salience in daily conversation, and (iii) using school literacy books. If this interpretation is correct, it motivates future research to understand more about these therapeutic activities, both those done in a clinical setting with a therapist, and those done outside of the clinic (including activities not traditionally seen as ‘therapy’, such as attending a stroke club).

Second, better outcomes in patients who received early clinical therapy were not the consequence of the therapy itself but instead reflect other factors which influence early therapy provision (or lack thereof). Patients may be prioritized over others for therapy if they either have greater potential to benefit, or better resources and/or social support in place to support their therapy (see Figure 1). For example, if a patient has better general health, greater motivation and attention, early signs of language improvement, or extra family support, therapists may use these assets to the patient’s advantage, capitalizing on their recovery potential. If those patients who received early therapy also enjoyed a positive therapeutic relationship, it may have further encouraged and enhanced the two-way engagement needed for successful rehabilitation (Lawton et al., 2018). On the other hand, patients who have poorer health, concomitant impairments, less motivation, or who are socially and/or technologically isolated may not be as able to engage in therapy as quickly, or may need rehabilitation from other professionals first – delaying speech and language therapy. These patients may also require further support to enhance their volition to engage with the therapy (Hart et al., 2019).

If this interpretation is correct, it highlights the need to understand in much greater detail the reasons why therapy may be prioritized or delayed – be they patient factors (e.g., cognitive, physical, psychosocial etc.) or service delivery factors (e.g., limited therapy resources, long waiting lists, logistics of reaching patients in the community etc.), see Figure 1. Whilst we already have some understanding of why patients do not receive the recommended amounts of therapy (medical instability, fatigue, administration and planning (Clarke et al., 2018)), we propose that a holistic and multidisciplinary understanding of speech therapy prioritization and resource allocation will provide new insights into the factors that need to be entered into predictive models of outcome after stroke (Godecke et al., 2013; Hope et al., 2013). This will enable better treatment planning, which keeps the patient at the core of the rehabilitation model and takes into account all of the factors which may influence their recovery.

Both of these speculative interpretations warrant further investigation, and highlight important directions for future studies. Regardless of which interpretation is correct, the early interaction between the patient and the therapeutic activity may harness and boost the neuroplastic changes that take place in the early weeks after stroke, thereby improving language outcomes beyond any spontaneous recovery (Nouwens et al., 2015) and leading to greater behavioural changes that are not lost in subsequent months.

### Limitations and future directions

As a retrospective analysis, it was impossible to control the many variables known to affect language outcomes at the point of entry into the study. We tackled this by factoring out the influence of many variables that are not usually controlled (e.g., initial severity and lesion size). However, other sources of variance may have biased the results as discussed below.

The first issue relates to the reliability of retrospective patient-reported outcome measures (PROMs) which depend on (i) the patient’s memory for the therapy received and the timing of their recovery; (ii) insight into own ability that might be more accurate for those who make a better recovery; and (iii) optimism or pessimism bias, such that patients who reported receiving more therapy may have reported better language outcomes due to recalling a positive experience with therapist input, rather than reflecting genuine language improvement. Recognizing the challenges posed by PROM data, we included objective measures of spoken language as well as the subjective measures, and demonstrated that Naming ability was better in those who did, versus did not receive early clinical therapy.

A second limitation is that we controlled variability measured in the chronic rather than acute stage post-stroke. Future studies would benefit from a detailed understanding of how the provision and benefits of early therapy depend on variables measured in the first week or two after stroke, including perceptual and cognitive impairments, initial severity of language symptoms, medical history, mental health, fatigue, attention, motivation, participation, education level, socioeconomic status (see Figure 1). This would help us to distinguish whether better outcomes in those who received therapy were the consequence of the therapy itself or a better potential to recover, or a combination of the two.

A third limitation is that, although we found no evidence that the Therapy and No Therapy groups differed in lesion size or damage to any part of the brain, it remains possible that those who made a poor recovery in the No Therapy group had a critical combination of damage that precluded recovery. This could be investigated in future by multivariate lesion analyses that are constrained by prior knowledge of the critical combination of damage that impedes recovery.

A fourth limitation is the lack of specific detail of therapy content, duration and frequency, done within and outside of the clinic. Patients’ estimates of their therapy may not reflect the amount of actual therapeutic input received. For example, a therapy session reported to last one hour may involve very few therapeutic activities targeting speaking impairments, e.g., if the therapist spent more time supporting psychological well-being. Further studies should i) focus on the proportion of time given to language-related therapeutic activities, rather than the less-exact ‘number of hours’ measure, ii) obtain more detail on the diverse contexts in which therapy may occur and (iii) ask patients to formally record their “therapy homework” and other language practice activities. By quantifying the number of specific therapeutic inputs, a measure of cumulative intervention intensity can be calculated (Brogan et al., 2020), and evaluated alongside all the other factors found to influence inter-patient variability in recovery (e.g., patients’ physical and mental health, cognitive abilities, level of engagement, demographics, social support). Ultimately, this will improve predictions of outcomes and the response to therapy at the individual patient level (Aguilar et al., 2018). Such studies would also offer a deeper insight into the therapeutic ingredients driving positive behavioural change and lead to more precise guidelines in the type, content and duration of the therapy recommended to stroke survivors with severe aphasia.

Finally, we note that, although we found that patients who received more hours of early therapy had better outcomes at one year post-stroke than those who received fewer hours, we are not able to infer what the optimal number of hours would be because no patients in our sample reported receiving more than 30 h of therapy in the first month.

## Conclusions

In a relatively large sample of stroke survivors with moderate-to-severe aphasia, those who reported receiving clinical speech and language therapy in the first month after their stroke achieved better long-term speaking abilities than those who reported not receiving therapy. This was observed after controlling for: time post-stroke at test, left and right hemisphere lesion size, initial severity of speaking impairment, amount of later therapy and speech comprehension ability. We suggest two, non-mutually exclusive, conclusions. The first is that early therapy may help patients develop self-management strategies that have a long-term benefit on their recovery. The second is that patients who receive more early clinical therapy have a better potential for recovery due to other influencing factors (Figure 1). If this conclusion is correct, we need to first understand the reasons why patients do and do not receive early therapy, and then devise a framework for measuring and entering these reasons into predictive models of individual-level outcomes and response to therapy. Our retrospective study also has implications for future therapy studies, and may inform directions for future trials: first by emphasising the factors that need to be controlled, second by identifying additional variables which may influence outcomes, and third by highlighting the benefits of combining objective behavioural assessments with patient-reported outcome measures of language function.

## Supplementary Material

Supplemental Material

## Data Availability

The data used in this study are stored in the PLORAS database (Seghier et al., 2016).
