# Supplementary material for: Better long-term speech outcomes in stroke survivors who received early clinical speech and language therapy: What’s driving recovery?
Source: Neuropsychol Rehabil. 2021 Jul 2;32(9):2319–41. doi: 10.1080/09602011.2021.1944883 (PMC12094264; doi:10.1080/09602011.2021.1944883)
Supplement: Supplemental Material [file PNRH_A_1944883_SM4410.pdf]

SUPPLEMENTAL MATERIAL

1. Supplemental literature review

1.1 Introduction

Prior studies of speech and language therapy for aphasia after stroke have yielded inconsistent evidence regarding its effects. This is because many factors thought to influence recovery need to be controlled, including therapy factors (e.g. timing of delivery, amount, and intensity), stroke factors (e.g. lesion size and site) and person factors (e.g. age and time post-stroke). Furthermore, there may be additional sources of variation in psychosocial factors such as motivation, or in domain-general cognitive processes such as attention. It is very challenging to control for all of these factors whilst securing sufficient statistical power for analysis. Therefore, despite the increasing research focus on aphasia therapy, it is difficult to compare trial results as they often differ in many of the factors described above, and/or do not have the necessary data available (e.g. imaging data).

We conducted a review of the currently available literature focusing on the effect of speech and language therapy, its intensity and the timing of its delivery. As a starting point, we examined Bhogal et al.'s highly influential review [(28)], which investigated the relationship between therapy intensity and change in language score for ten different therapy trials. They observed that the 5 "positive" trials that demonstrated a significant impact of therapy provided an average of 8.8 hours per week for an average of 11.2 weeks (averaging 98 hours in total per patient), whereas the 5 "negative" trials that failed to demonstrate significant therapy effects provided an average of 2 hours per week for an average of 22.9 weeks (averaging 44 hours in total per patient). From this, they concluded that high-intensity therapy is more beneficial than low-intensity therapy. Critically, however, the "positive" versus "negative" trials were not matched for lesion site, initial severity, time post-stroke, or therapy type. Regarding timing of therapy, our literature search did not reveal any individual study that directly compared the effect of early- versus late-initiated therapy on speaking outcomes, although a between-study meta-analysis of 55 therapy studies by Robey [(29)] concluded that therapy outcome was better in studies that provided earlier than later therapy. Nonetheless, there were, again, multiple uncontrolled factors across the meta-analysed studies, including initial symptom severity.

Unlike prior reviews [(1, 25, 28-30)], our review: (1) selected studies that compared speaking recovery in at least two groups of stroke survivors who primarily differed in whether or not they had received speech and language therapy or in the intensity of therapy; (2) split the studies according to whether the therapy was administered in the acute period (within the first 4 weeks) or sub-acute period (after the first 4 weeks), allowing us to explore therapy intensity effects at different time-points; and (3) analysed the evidence from 7 new studies that were not yet published at the time of the 2016 Cochrane systematic review of speech and language therapy for aphasia following stroke [(1)]. Although the studies we reviewed differed from one another in terms of the specific type of speech and language therapy administered, speaking

ability was consistently defined as one of the outcome measures. The results are discussed below, with further details in Tables I and II.

1.2 Methods

Our primary source of therapy trials was the 2016 Cochrane systematic review of speech and language therapy for aphasia following stroke [(1)]. We also explored PubMed and Web of Science, using search terms such as aphasia/anomia, therapy/rehabilitation/treatment, intensity/duration/amount, timing/early/acute/sub-acute. Reference lists of relevant papers yielded further studies. The inclusion criteria were: i) at least two therapy intensities compared, one of which could be no therapy; (ii) sufficient numbers of participants to allow the significance of therapy effect sizes to be evaluated (all single case studies were excluded), (iii) aphasia caused by stroke, not neurodegeneration (e.g. primary progressive aphasia); and (iv) changes in speaking ability were part of the outcomes measured. We found 15 studies that met these inclusion criteria (Table I), including seven published since the 2016 Cochrane systematic review. No trials were found that directly compared the effect of early- versus late-initiated therapy on speaking outcomes. Unlike the Cochrane systematic review, we categorised studies according to when the therapy was administered: early (within the first 4 weeks of stroke onset) or later (more than 4 weeks post-onset), allowing us to explore therapy intensity effects at different time-points, and to extend the discussion offered by the Cochrane systematic review.

1.3 Early therapy effects

We identified a total of 8 studies that compared the effect of early (in the first month) speech and language therapy to no therapy or lower amount of therapy. Four of these studies showed a significant advantage of therapy compared to no or very little therapy (see Table 2). However, their conclusions are weakened by a number of factors. The first two studies, both by Godecke et al. [(2, 3)], report that initial severity was worse in the low-intensity therapy group than the high-intensity groups. More critically, a longitudinal follow-up of outcome at 6 months in patients from the first of these studies [(2)] revealed non-significant results suggesting that better outcomes in the high-intensity therapy group were lost over time. The third study, by Mattioli et al. [(31)] used a very small sample size (only 6 patients per group), and did not control for all the non-therapy factors that influence recovery. The fourth study, by Nouwens et al. [(32)], only observed significant effects of therapy (versus no therapy) after removing 71% of the participants who were unable to complete the intervention. The authors therefore noted that the therapy advantage should be interpreted with great caution, because patients who were unable to tolerate intensive therapy were only excluded from the therapy group. When the groups were better matched, speaking outcome did not differ for the therapy versus no therapy groups.

No significant advantage of early high- versus low-intensity therapy was found in the remaining four studies. The fifth study, by Laska et al.[(33)], compared no therapy to 3.75 hours per week of therapy that predominantly targeted language comprehension. Unsurprisingly, this resulted in no significant benefits on speech production, the primary outcome. The sixth study, by Bowen et al. [(20)], provided standard clinical therapy at a lower intensity (1.3 hours per week) than those studies by Godecke et al. [(2)], Mattioli et al. [(31)], and Nouwens et al. [(32)], which found

significant therapy effects (2.5, 5 or 7 hours per week). Moreover, because this was typical clinical therapy delivered through the UK National Health Service, the 1.3 hours of speech and language therapy per week included broader contact time (including, for example, multidisciplinary team planning) rather than just intervention time. It may therefore be possible that the amount of therapy delivered to their patients was not enough to result in a detectable improvement. The seventh study, by Woldag et al. [(34)], found no significant advantage of 15 hours per week of Constraint-Induced Aphasia Therapy (CIAT) compared to 7 hours per week of conventional therapy. This outcome led the authors to conclude that 7 hours of conventional communication therapy was as effective as 15 hours of CIAT but there was no demonstration of the effectiveness of 7 hours of conventional communication therapy compared to no therapy. The eighth study, by Godecke et al. [(4)], was a randomised controlled trial borne from the positive significant effect of early therapy found in pilot trials (described above,[(2, 3)]). Contrary to their expectations, the authors found no significant advantage of early intensive therapy (5 hours per week) compared to usual care (2.3 hours per week). Whilst both patient groups improved over time, the lack of a no-therapy control makes it impossible to infer that the improvement was the consequence of therapy.

It is also relevant to note that sample size was at least twice as large in studies showing non-significant intensity effects as studies showing significant intensity effects. For example, the studies with non-significant therapy intensity effects by Laska et al., Bowen et al. and Godecke et al. [(33, 20, 4)] included 60-147 participants per group, whereas only 6-30 participants per group were included in the studies with significant therapy intensity effects by Godecke et al., Mattioli et al., and Nouwens et al [(2, 3, 31, 32)]. This raises two possibilities: first, that group differences might be confounded by differences in the patients' potential for spontaneous recovery, and second, that the influence of outliers is greater where sample sizes are smaller. Both of these can be better controlled with larger sample sizes.

In summary, the evidence for the benefit of early therapy is currently weak. Inconsistent conclusions across studies might reflect discrepancies in (i) what constitutes a high versus low intensity therapy; (ii) participant numbers, (iii) how well groups are matched for non-therapy factors that are known to influence recovery (e.g. initial severity and lesion site), (iv) the criteria used to determine therapy time and (v) the type of therapy administered.

1.4 Later therapy effects

We identified another 7 studies that compared high- versus low-intensity speech and language therapy at a later stage post-stroke (i.e. >one month after onset); only one of which, by Breitenstein et al. [(35)], showed a significant benefit of high-intensity therapy. This was a well-powered study, which reported a medium effect size (Cohen's d = 0.58), with 78 participants per group, conducted 2 to 3 years after stroke for a period of 4-5 weeks, with 10 hours or more per week of therapy in the high-intensity group and 1.5 hours per week of therapy in the low-intensity group.

Five of the other 6 studies (that did not find a significant therapy effect) had (i) fewer participants (9-18 per group) and (ii) a higher amount of therapy in the low intensity therapy group [1.8-2.5, 2, 3, 6, 6 hours per week). These were reported by Dignam et al., Kesav et al., Denes et al., Martins et al., and Stahl et al [(36-40)]. The remaining study, by Bakheit et al. [(41)], included more participants per group

(approximately 50 in each) but the high therapy intensity (4.3 hours per week) was less than half that of the Breitenstein study [35] showing a significant effect (10 hours or more per week).

Contrary to expectation, two studies of later therapy (by Kesav et al. and Dignam et al.) found that the low-intensity intervention was significantly more beneficial than the high-intensity intervention. Specifically, better speaking outcomes were reported after 3 hours per week than 6 hours per week of therapy in Kesav et al. [37] and 6 hours per week than 16 hours per week of therapy in Dignam et al. [36]. These findings suggest that too much therapy is at best unnecessary, and at worst, disadvantageous in sub-acute or chronic stroke patients, for example, when the benefits of high-intensity therapy are outweighed by the higher dropout rate [1, 41]. Furthermore, there may be a "therapy ceiling effect", which, if reached, may result in patients ceasing to improve, and potentially even declining [40].

1.5 Summary

Overall, our review of studies that compared high- versus low-intensity speech and language therapy in chronic stroke patients only found inconclusive evidence that high-intensity therapy is beneficial. The strongest results come from Breitensten et al. [35] who administered their therapy 2-3 years after stroke. The results of early therapy studies were more consistent but did not provide evidence for long-term maintenance of the early therapy benefit. As already mentioned above, we did not identify any studies that directly compared the effect of early- versus late-initiated therapy on speaking outcomes although a review by Robey [29] suggested that early therapy might be more beneficial.

Table I. Therapy trials reviewed.

Therapy commenced within the first month post-stroke	
Study reference	Study
[2]	Godecke et al., 2012
[3]	Godecke et al., 2014
[31]	Mattioli et al., 2014
[32]	Nouwens et al., 2017
[33]	Laska et al., 2011
[20]	Bowen et al., 2012
[34]	Woldag et al., 2017
[4]	Godecke et al., 2020
Therapy commenced after the first month post-stroke	
[35]	Breitenstein et al., 2017
[36]	Dignam et al., 2015
[37]	Kesav et al., 2017
[38]	Denes et al., 1996
[39]	Martins et al., 2013
[40]	Stahl et al., 2017
[41]	Bakheit et al., 2007

Table II. Results of therapy trials comparing hours of therapy on speaking outcomes.

Study	Therapy onset	Therapy duration	Low-intensity		High-intensity		Best therapy intensity
			Hours per week	*N*	Hours per week	*n*	
	Days since stroke	Weeks	Acute period (therapy onset within one month of stroke).				
(2)*	3	4	<0.25	27	2.5	32	High
(3)*	6	Low=3 High=4.5	<0.25	27	4.25	20	High
(31)	2	2	0	6	5	6	High †
(32)	12	4	0	72	7	23	High ‡
(33)	3	4	0	61	3.75	62	Neither
(20)	15	16	0	72	1.3	81	Neither
(34)	19 §	2	7	20 | |	15	20	Neither
(4)	8-10	4	2.3	70	5	147	Neither
	Months since stroke	Weeks	Sub-acute/chronic period (therapy onset > 4 weeks post-stroke)				
(35)	27-43	Low=4 High=4.8	1.5	78	10	78	High #
(36)	31–47	Low=8 High=3	6	18	16	16	Low
(37)	1 §	4	3	9	6	11	Low
(38)	3	24	1.8 - 2.5	9	3.75 – 5	8	Neither
(39)	2 §	Low=50 High=10	2	9	10	9	Neither
(40)	65	4	6	15	12	15	Neither
(41)	1 §	12	1.6	46	4.3	51	Neither

* Studies (2) and (3) are not independent of one another: they used the same control group.
† Therapy benefit was maintained at 6-month follow-up.
‡ A further 57 participants were included in the High-intensity group who did not complete the full 7 hours per week. There was no significant benefit of either therapy intensity in this analysis.
§ Did not control for spontaneous recovery.
| | A third group *[n=20]* received 15 hours per week of conventional communication therapy (to compare with the same intensity of Constraint Induced Aphasia Therapy received by the first intervention group); CIAT resulted in significantly better outcomes than conventional therapy (same intensity).
The control group made similar improvements after receiving the same intensity of therapy as the intervention group after a 3-week deferral period.

Supplemental Figure 1. Correlation matrix

	EThH	EThG	LThH	LH	RH	Sev	Age	Tps	SM	AWC	Hand
**EThH**	1	0.649**	0.263**	0.016	-0.063	0.023	-0.1	0.034	0.091	-0.057	0.135
**EThG**	0.649**	1	0.148	0.032	-0.109	0.022	-0.021	0.047	-0.002	-0.084	0.265**
**LThH**	0.263**	0.148	1	0.255**	-0.144	-0.113	-0.246**	0.097	0.042	-0.12	-0.06
**LH**	0.016	0.032	0.255**	1	0.05	-0.23**	-0.066	0.193*	-0.185*	-0.35**	0.03
**RH**	-0.063	-0.109	-0.144	0.05	1	-0.024	0.215*	-0.06	-0.457**	-0.249**	-0.019
**Sev**	0.023	0.022	-0.113	-0.23**	-0.024	1	0.068	-0.026	0.091	0.214*	0.116
**Age**	-0.1	-0.021	-0.246**	-0.066	0.215*	0.068	1	-0.242**	-0.246**	-0.246**	0.013
**Tps**	0.034	0.047	0.097	0.193*	-0.06	-0.026	-0.242**	1	0.106	0.052	-0.068
**SM**	0.091	-0.002	0.042	-0.185*	-0.457**	0.091	-0.246**	0.106	1	0.426**	-0.014
**AWC**	-0.057	-0.084	-0.12	-0.35**	-0.249**	0.214*	-0.246**	0.052	0.426**	1	0.016
**Hand**	0.135	0.265**	-0.06	0.03	-0.019	0.116	0.013	-0.068	-0.014	0.016	1

Abbreviations: EThH = Early therapy hours, EThG = Early therapy group, LThH = Later therapy hours, LH = left hemisphere lesion size, RH = right hemisphere lesion size, Sev = initial severity, Age = age at stroke, Tps = time post-stroke of CAT, SM = Semantic memory, AWC = auditory word comprehension, Hand = handedness

* = correlation is significant at 0.05 level

** = correlation is significant at 0.01 level
